# Wearable and Non-Invasive Sensors for Rock Climbing Applications: Science-Based Training and Performance Optimization

**DOI:** 10.3390/s23115080

**Published:** 2023-05-25

**Authors:** Miyuki Breen, Taylor Reed, Yoshiko Nishitani, Matthew Jones, Hannah M. Breen, Michael S. Breen

**Affiliations:** 1 Department of Mathematics, North Carolina State University, Raleigh, NC 27695, USA; mbreen@alumni.ncsu.edu; 2 The Beta Angel Project, Alexandria, VA 22304, USA; taylor@beta-angel.com (T.R.); hmsbreen@gmail.com (H.M.B.); 3 Sportrock Performance Institute, Alexandria, VA 22304, USA; 4 Rikkyo Research Institute of Wellness, Rikkyo University, Tokyo 171-8501, Japan; yoshiko_nis@yahoo.co.jp; 5 Jones Fitness and Performance, Charleston, SC 29412, USA; jonesfitnessandperformance@gmail.com; 6 Eno River Academy, Hillsborough, NC 27278, USA; 7 Department of Civil, Construction, and Environmental Engineering, North Carolina State University, Raleigh, NC 27695, USA

**Keywords:** wearable sensors, non-invasive sensors, biomonitoring, cardiac sensors, breathing sensors, physical sensors, external and embedded sensors, rock climbing, sport climbing, bouldering, speed climbing, outdoor climbing

## Abstract

Rock climbing has evolved from a method for alpine mountaineering into a popular recreational activity and competitive sport. Advances in safety equipment and the rapid growth of indoor climbing facilities has enabled climbers to focus on the physical and technical movements needed to elevate performance. Through improved training methods, climbers can now achieve ascents of extreme difficulty. A critical aspect to further improve performance is the ability to continuously measure body movement and physiologic responses while ascending the climbing wall. However, traditional measurement devices (e.g., dynamometer) limit data collection during climbing. Advances in wearable and non-invasive sensor technologies have enabled new applications for climbing. This paper presents an overview and critical analysis of the scientific literature on sensors used during climbing. We focus on the several highlighted sensors with the ability to provide continuous measurements during climbing. These selected sensors consist of five main types (body movement, respiration, heart activity, eye gazing, skeletal muscle characterization) that demonstrate their capabilities and potential climbing applications. This review will facilitate the selection of these types of sensors in support of climbing training and strategies.

## 1. Introduction

Progress in sport science has been driven by data. Wearable sensors/wearables (i.e., body-worn sensors such as accelerometers, heart monitors) can capture detailed, continuous, and objective measurements of our physical activity. The global wearable sensors market is projected to reach USD 5.2 billion by 2028, growing at 29% annual growth rate from 2021 to 2028 [1]. Such worldwide proliferation of wearable sensors provides unprecedented opportunities for the collection of data to study and improve sport performance. With small and lightweight designs, large storage, rapid processors, and wireless transmission, wearable sensors can collect a tremendous amount of data. Without sacrificing performance, wearable sensors and other types of non-invasive sensors enable us to obtain data during a sport activity. These data provide valuable science-based information and insights, which can be used by participants, coaches, and scientists to enhance training and develop strategies for various sports including rock climbing. The data-driven sport science approaches have been applied in an effort to determine what distinguishes high-performance athletes from others, such as the study conducted with a world-renowned professional rock climber, Adam Ondra [2].

Rock climbing is a modern discipline in sports, which has grown rapidly both nationally and internationally, along with tremendous advances in the climbing equipment and indoor climbing gym markets. In 2022, there were 500 indoor climbing gyms in the US with a USD 493 million industry. According to the International Federation of Sport Climbing (IFSC), there are 44.5 million climbers worldwide [3]. Competitive climbing debuted at the 2020 Tokyo Olympics as a new sport with 40 athletes, and accepted officially at the 2024 Paris Olympics with a substantial expansion in the number of athletes to 68 (70% increase) [4]. 

Rock climbing has some unique requirements that are different than other traditional sports (e.g., soccer, ice-hockey). First, climbing requires efficient body movements to maintain the energy to ascend difficult climbing routes [5]. Second, climbing requires high strength and endurance from smaller muscles (e.g., forearm and finger flexing muscles) as compared to the larger muscles used during running [6]. Third, climbing requires high localized muscle oxygen demand (e.g., forearm muscles) and less systemic oxygen demand that is needed for cardio-intensive sports [6]. Fourth, climbing can induce psychological stress due to potential falls from high elevations [7]. Finally, climbing requires a high visual–motor skill level to visually explore and coordinate body movements to ascend new bouldering and lead climbing routes that are unknown to the climber and cannot be rehearsed before competitions [8]. This is unlike traditional sports (e.g., soccer, tennis) that are played on a standardized field. 

Based on these unique requirements, rock climbing can benefit from several types of biometric information during climbing. This biometric information needs to be continuous time-course measurements that include: biomechanical (e.g., body movement), physiological (e.g., heart activity, respiration, skeletal muscle characterization), cognitive/mental (e.g., eye gazing during exploration of climbing route), and psychological (e.g., stress) responses during climbing. Furthermore, this biometric information can be further enhanced with spatial information about the location of the climber relative to spatially varying characteristics of the climbing route (e.g., video scene cameras).

There are two main classes of sensors used in rock climbing: (1) class 1 includes wearable and non-invasive sensors that can continuously collect data during climbing with minimal or no impact on climber, and (2) class 2 includes traditional sensors (e.g., dynamometers) and other sensors that cannot collect data during climbing. Class 1 sensors can be separated into three categories: wearable, external, and embedded. External sensors are mounted at fixed, strategic locations in the climbing environment (e.g., camera at base of climbing wall). Embedded sensors are electronic devices integrated into climbing-specific equipment used during climbing (e.g., force sensors embedded within holds on indoor climbing walls). Class 2 sensors, such as traditional dynamometers that assess the strength of climbing-specific muscles, are the simplest and most common type of sensors used in climbing. Dynamometers measure the mechanical force generated by specific muscles when a climber squeezes or pulls a sensing device. However, these sensors are limited due to their inability to collect data during climbing. 

This review paper focuses on class 1 sensors (i.e., wearables, external, embedded) used during climbing. Class 1 sensors collect detailed biometric data to determine biomechanical (e.g., body movement), physiological (e.g., heart rate), and mental (e.g., route exploration) responses during climbing, which can be used to derive indicators of climbing performance (e.g., movement fluency, physical exertion, mental acuity). These sensors enable data-driven approaches for training, strategies, and performance optimization.

This paper provides an overview and critical analysis of the scientific literature on several selected class 1 sensors to demonstrate their capabilities and potential climbing applications. The review includes five types of sensors: (1) body movement; (2) respiration; (3) heart activity; (4) eye gazing; and (5) skeletal muscle characterization. Below, we first provide an overview of climbing and the five types of sensors. We then highlight climbing studies that applied these sensors, and describe the benefits, limitations, and practical considerations for selecting the sensors. 

## 2. Overview of Climbing

**Figure 1:**
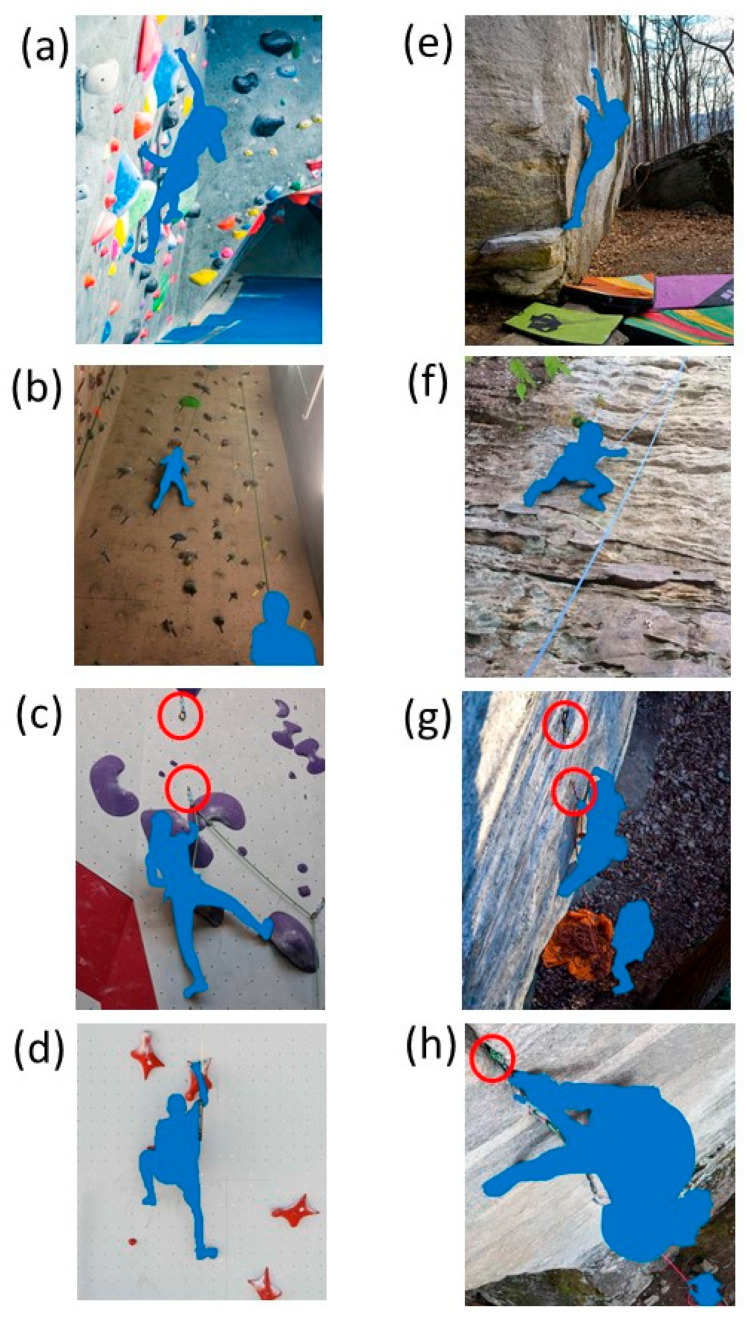
Types of indoor (**a**–**d**) and outdoor (**e**–**h**) rock climbing. The four types of indoor climbing include bouldering (**a**), top rope (**b**), sport lead (**c**), and speed climbing (**d**). The four types of outdoor climbing include bouldering (**e**), top rope (**f**), sport lead (**g**), and traditional (trad) lead climbing (**h**). Indoor bouldering (**a**) shows a blue padded floor below the climber. Indoor top rope climbing (**b**) shows the rope path that goes above the climber to a top anchor and then towards the floor to a belayer below. Indoor lead climbing (**c**) shows anchors (red circles) along the climbing route. Indoor speed climbing (**d**) shows the rope path that goes above the climber to a top anchor. Outdoor bouldering (**e**) shows several ground pads below the climber. Outdoor top rope climbing (**f**) shows the rope path that goes above the climber to a top anchor and then towards the ground to a belayer below. Outdoor lead climbing (**g**) shows anchors (red circles) along a climber’s route. Outdoor trad lead climbing (**h**) shows a removable anchor (red circle) inserted in a large crack in the rock near a climber.

Indoor climbing uses hand and foot holds (e.g., constructed from polyurethane, fiberglass) attached to walls (e.g., plywood) orientated at various angles. There are four main types of indoor climbing: bouldering, top rope, sport lead, and speed (Figure 1). Bouldering is performed on short walls (typically 4–5 m) with floor pads used for protection during a fall. Top rope, lead, and speed climbing are performed on tall walls (typically 15–20 m) with a rope and wall anchors used for protection during a fall. One end of the rope is attached to the climber, and the other end is attached to a belayer on the floor. For top rope climbing, the rope is attached to an anchor at the top of the wall prior to the climb, and the belayer removes slack in the rope as the climber ascends. For lead climbing, the climber places the rope through permanent anchors along the climbing route while ascending, and the belayer adjust slack in the rope as the climber ascends. The goal of bouldering, top rope, and lead climbing is to ascend various types of climbing routes without falling. Speed climbing is a specialized version of top rope climbing where the goal is to ascend a standardized 15 m climbing route in the fastest way. 

Outdoor climbing, which uses natural rock formations, consists of four main types of climbing: bouldering, top rope, sport lead, and traditional (trad) lead climbing (Figure 1). For outdoor bouldering, top rope, and sport lead climbing, the climbing method is the same as the corresponding indoor method previously described. Trad lead climbing is a specialized version of lead climbing where the climber places the rope through removable anchors. These removable anchors are inserted into cracks and other rock features along the climbing route while ascending, whereas sport lead climbing uses permanent anchors bolted into the rock.

There are several key performance indicators for climbing. First, climbing fluency is an indicator describing the ease and efficiency of movement during climbing, which corresponds to climbing skill [5]. Second, breathing rate during climbing is an indicator of perceived exertion and fatigue [9]. Third, heart rate can be used an indicator of psychological factors related to climbing (e.g., stress from fear of falling) [7]. Fourth, skill level for visual inspection of a climbing route is an indicator of climbing fluency and performance [10]. Finally, activity and functional status of the forearm flexor muscles during climbing are key limiting factors for climbing performance [11]. These performance indicators can be assessed using sensors during climbing. 

## 3. Types of Sensors

**Figure 2:**
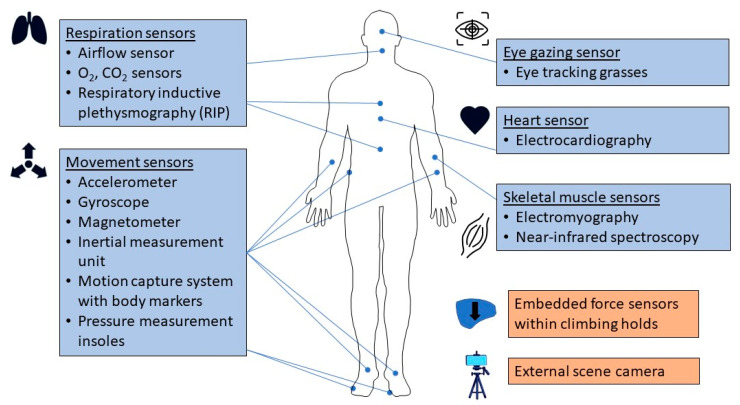
Sensors used during climbing. The five types of sensors are respiration, movement, eye gazing, heart, and skeletal muscles, which consist of wearable sensors (blue boxes), embedded force sensors and external scene cameras (tan boxes).

Figure 2 shows the five types of sensors: body movement, respiration, heart activity, eye gazing, and skeletal muscle conditions. 

### 3.1. Body Movement Sensors

**Figure 3:**
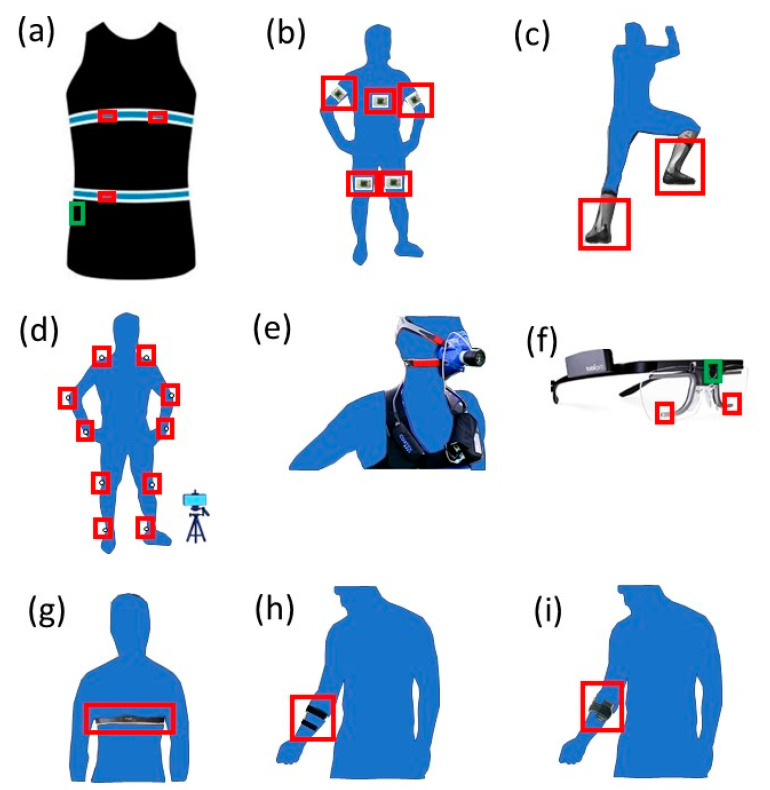
Examples of sensors used during climbing. Movement sensors: (**a**) Hexoskin biometric shirt with accelerometer inside hip pocket (green), (**b**) MotionPod IMU attached to various body segments (red), (**c**) Pedar-X force sensors within shoe insoles (red), (**d**) motion capture system with external camera, reflective body markers (red). Respiration sensors: (**a**) Hexoskin biometric shirt with thoracic and abdominal chest band displacement sensors (blue), (**e**) METAMAX 3B with face mask and chest-mounted data collection system. Eye gazing sensor: (**f**) Tobii Pro Glasses with eye tracking cameras (red) and scene camera (green). Heart sensors: (**a**) Hexoskin biometric shirt with three flexible ECG sensors (red), (**g**) Polar monitor with chest band ECG sensors (red). Skeletal muscle sensors on forearm (red): (**h**) EMG electrodes, (**i**) PortaMon with NIRS sensor.

Body movement sensors are used to measure the biomechanical movement during climbing (Figure 3). These sensors include inertial measurement units (IMU), standalone accelerometers, pressure measurement insoles, three-dimensional motion capture systems (MCS), and force sensors embedded with indoor climbing holds (Table 1).

The IMU devices consist of three types of integrated sensors: accelerometer, gyroscope, and magnetometer. The accelerometer measures the rate of change in velocity (i.e., gravitational force) along the three linear and orthogonal directions (e.g., x, y, z). The gyroscope measures the rate of change in rotational motion (deg/s) around the x, y, z axes. The magnetometer measures the earth’s magnetic flux density (T) in the x, y, z directions to determine the sensor direction in an earth reference frame. A key benefit of IMU devices, is the ability to continuously measure linear and rotational movements at different body joints (e.g., elbows, knees, hips) using multiple time-matched IMU devices with each IMU attached to a different body segment (e.g., forearm, upper arm, lower leg, upper leg, hip, torso). Seifert et al. used an IMU device (MotionPod, Movea, Grenoble, France) attached to the hip during climbing to determine the smoothness of hip trajectories and orientations [12,13].

**Figure 4:**
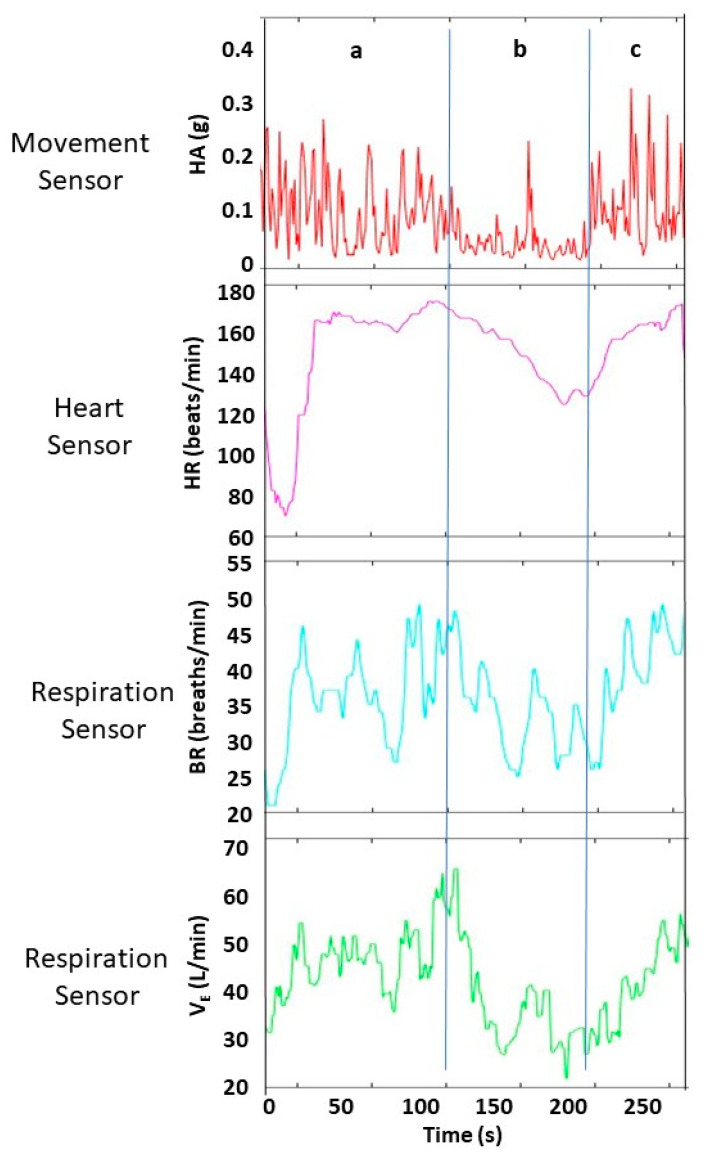
Examples of raw data collected during lead climbing from three types of wearable sensors (movement, heart, respiration). Data are shown across three consecutive periods (**a**–**c**) with different climbing activities (Breen 2022, [14]). First period (**a**) corresponds to climber interacting with climbing surface (e.g., hold change, rope clipping) that shows increasing heart rate (HR), breathing rate (BR), minute ventilation (V_E_) and many large peak-to-valley changes in hip acceleration (HA). Each HA peak often corresponds to a hold change. Second period (**b**) corresponds to immobility (e.g., resting, route finding) and stationarity (e.g., chalking, limb shaking) that shows decreasing HR, BR, V_E_, and mostly small peak-to-valley changes in HA. Third period (**c**) corresponds to climber interacting with climbing surface (e.g., hold change, rope clipping) that shows characteristics similar to period (**a**).

Standalone accelerometers, which are IMU devices without a gyroscope or magnetometer sensors, provide data that are easier to interpret than IMU and not susceptible to possible drift errors from gyroscopes and magnetometers. Breen et al. used a biometric compression shirt (Hexoskin, Carre Technologies Inc., Montreal, QC, Canada) (Table 1) to measure hip acceleration during climbing [14,15]. The accelerometer is embedded within a data logger, which is secured in a sewn-in pouch in the compression shirt near the right hip. The triaxial accelerometer continuously acquires x, y, and z accelerations at 64 Hz, which determine the magnitude of hip acceleration averaged across 1 s. During a lead climb, hip accelerations showed large transient spikes (maximum = 0.36 g) with short periods of sustained lower accelerations (0.01–0.08 g) (Figure 4). Using time-matched climbing video, the large spikes correspond to ascending movement, and the sustained lower values correspond to resting. During a lead climb fall, the acceleration = 1.0 g, which indicates the values are reasonable. Villar et al. showed a mean absolute error of 2% [16].

Pressure measurement insoles are sensors embedded within a shoe insole that measure vertical force loading on foot holds during climbing. Balas et al. used a pressure distribution insole (Novel Pedar X, Munich, Germany) during climbing [17,18]. Each insole contains a two-dimensional spatial array of 99 force sensors with a 10 mm spatial resolution and a 15–600 N dynamic range. The insole connects to a waist-mounted data logger. For each foot, the insole measures the vertical force loading (N) for each sensor at 100 Hz. Using time-matched climbing video, a force-time integral for each foot hold on a climbing wall was calculated using force data from all sensors and normalized by body weight. Across three top rope climbing routes (−5, 0, +3° overhanging), the vertical loading on the foot holds was 2624 ± 44 N s/kg (mean ± SD).

**Table 1:** Overview of sensors used for climbing studies.

Sensor Type	Sensor Location	System Details	Measurement	Product (Company)	Reference
Body movement—accelerometer	Hip	Biometric shirt with accelerometer in hip shirt pocket	Triaxial linear acceleration (g)	Hexoskin (Carre Technologies, Montreal, QC, Canada)	[14]
Body movement—IMU (accelerometer, gyroscope, magnetometer)	Arms, legs, chest	Device with three time-matched sensors	Triaxial linear acceleration (g), rotational velocity (rad/s), earth magnetic field (T)	MotionPod (Movea, Grenoble, France)	[19,20,21]
Body movement—insole pressure distribution	Insole of shoe	2D spatial array of force sensors (10 mm resolution)	Vertical force between foot and shoe (Pa)	Pedar-X (Novel, Munich, Germany)	[17]
Body movement—motion capture system (MCS)	Visible body markers	Multiple synchronized video cameras to capture 3D spatial location	Center of gravity horizontal distance from wall (m), velocity (m/s)	Mac 3D System (Motion Analysis, Santa Rosa, CA, USA)	[22]
Body movement—force sensor embedded within climbing hold	Embedded within holds on climbing wall	Force sensors within climbing holds with wireless data transfer	Triaxial linear force (Pa)	K3D120 triaxial force sensor (ME-Meβsysteme, Hennigsdorf, Germany)	[23]
Respiration—airflow	Face mask	Airflow sensor within breathing apparatus, chest/backpack unit	Minute ventilation (L/min)	METAMAX 2B (Cortex, Biophysik, GmbH, Leipzig, Germany)	[24]
Respiration—O_2_, CO_2_	Face mask	O_2_, CO_2_ sensors within breathing apparatus, chest/backpack unit	O_2_ consumption (L/min) CO_2_ release (L/min)	METAMAX 2B (Cortex, Biophysik, GmbH, Leipzig, Germany)	[24]
Respiration—respiratory inductive plethysmography (RIP)	Chest bands	Biometric shirt with RIP sensors in two chest bands	Breathing rate (breaths/min), minute ventilation (L/min)	Hexoskin (Carre Technologies, Montreal, QC, Canada)	[14]
Heart activity—electrocardiography (ECG)	Chest	Biometric shirt with three flexible ECG sensors	Heart rate (beats/min)	Hexoskin (Carre Technologies, Montreal, QC, Canada)	[14]
Heart activity—electrocardiography (ECG)	Chest	ECG sensors attached to chest strap	Heart rate (beats/min)	Polar (Polar Electro OY, Kempele, Finland)	[24]
Eye gazing—eye tracking glasses (ETG)	Face	Cameras embedded within eye glass frame	Eye gaze patterns toward holds on climbing wall	Tobii Pro Glasses 2 (Tobii, Stockholm, Sweden)	[8]
Skeletal muscle—electromyography (EMG)	Forearms	Skin surface EMG sensors attached to local muscle	Electrical activity of muscle	Tel-100 System (BioPac Systems Goleta, CA, USA)	[25]
Skeletal muscle—near infrared spectroscopy (NIRS)	Forearms	Skin surface near-infrared light source and detector	Muscle oxygen saturation = oxy-hemoglobin/total hemoglobin	PortaMon (Artinis Medical Systems, BV, Zetten, The Netherlands)	[26]

MCS can determine 3D spatial information during climbing. There are two main types of MCS: marker-based and marker-less systems. Marker-based systems use markers attached to a climber and multiple synchronized scene cameras. The markers are attached to different body locations that are visible in the climbing videos. Nishitani et al. 2008 used an MCS to compare body movement differences between skilled and beginner climbers [22]. Reflective markers were attached to the climbers at several body locations (ankles, knees, hips, elbows, wrists, head). During climbing, the spatial information of the markers was measured using 12 cameras and a 3D motion analysis system (Mac 3D system, Motion Analysis Co.) [27]. The images from the multiple cameras were combined into 3D images. The MCS determined the time-course plots of the horizontal distance between the climber’s center of gravity and the wall, speed of the center of gravity, and force of the center of gravity. The analysis, which focused on a high-step body movement for bouldering, showed that skilled climbers reduced stress on upper limbs and fingers with three unique climbing characteristics: having the body closer to the wall, shorter duration for the pulling stage, and smaller force needed during the retention stage.

Marker-less MCS use computer vision software and video from one or more cameras to determine the orientation and position of various body joints without attaching markers to a climber. Without markers, the system setup can be simpler but less accurate than marker-based systems. Pandurevic et al. used an MCS to determine the body joint angles, and the distance between the body’s center of gravity and the wall during bouldering [23]. The system included a depth camera (Realsense D435, Intel, Santa Clara, CA, USA) and data analysis software (OpenPose) [28]. The system created animations of the climber’s joint angles, and time-course plots of the distance from the wall, which varied between 0.20 and 0.55 m during climbing. 

Triaxial force sensors embedded with climbing holds are used to measure magnitude and direction of forces applied to foot and hand holds, and the associated distribution of body weight on hands versus feet. Pandurevic et al. used a wireless force measurement system embedded within eight climbing holds to create a bouldering problem on an indoor climbing wall [23]. The instrumented holds contained a triaxial force sensor, battery powered microcontroller and WiFi module for wireless data transfer, and synchronized force measurements. Using time-matched climbing video, time-course plots of the ratio of force between hands and feet showed variations between 0.2 and 1.4, where ratios > 1 indicates greater force from hands as compared to feet. 

### 3.2. Respiration Sensors

Respiration sensors are used to measure breathing-related biometrics during climbing. These respiratory sensors include wearable metabolic systems and respiratory inductive plethysmography (RIP) systems (Figure 3, Table 1), which provide a direct and indirect methods, respectively, to determine breath-by-breath respiratory parameters.

Wearable metabolic systems consist of a face mask with integrated airflow and gas (O_2_, CO_2_) sensors. Balas et al. used a metabolic system (Metamax 3B Cortex, Leipzig, Germany) during climbing to determine whole-body systemic O_2_ uptake (VO_2_), CO_2_ production (VCO_2_), respiratory exchange ratio (RER = VCO_2_/VO_2_), minute ventilation (V_E_), breathing rate (BR), and tidal volume (V_T_) [26,29]. The face mask is designed with a bidirectional digital turbine to determine inhaled and exhaled air volumes. A sampling tube is attached to the turbine to measure O_2_ and CO_2_ concentrations using an electrochemical cell and infrared analyzer, respectively. During exhaustive incremental climbing tests, the peak V_E_, V_T_, and BR were 97.5 ± 15.6 L/min, 47 ± 6 L, and 2.3 ± 0.3 breaths/min (mean ± SD), respectively; and peak VO_2_ and RER were 45.3 ± 4.6 mL/min/kg and 1.07 ± 0.11 (mean ± SD), respectively. 

Wearable RIP systems consist of chest bands with embedded sensors that continuously measure inspiration and expiration displacements of the bands. Breen at al. used a biometric compression shirt (Hexoskin, Carre Technologies Inc., Montreal, QC, Canada) to continuously measure V_E_ and BR during climbing [14]. These respiratory metrics are estimated using two strain gauges sewn directly into the shirt fabric (one at the thoracic level, one at the abdominal level), which collect chest band displacements at 128 Hz. Elliot et al. showed mean absolute errors of 3% for BR, and 5–8% for V_E_ during moderate-intensity exercise (50–75% maximum work rate during stationary cycling) [30]. Villar et al. showed mean absolute errors of 2% for BR during moderate- and high-intensity exercise [16]. 

Using the Hexoskin compression shirt during lead climbing, Breen et al. showed large variability of V_E_ and BR with a range (min-max) of 22–67 L/min and 21–53 breaths/min, respectively [14]. Using time-matched climbing video, Figure 4 shows time-course measurements of BR and V_E_ during three climbing stages: ascending (stage 1), resting on the wall (stage 2), then continued ascending (stage 3). During stage 1, BR rapidly increases from 22 to 48 breaths/min, and then fluctuates between 28 and 50 breaths/min. During stage 2, BR decreases overall. During stage 3, BR increases. For stages 1–3, V_E_ showed a similar temporal pattern as compared to BR. 

### 3.3. Heart Activity Sensors

Wearable heart sensors measure the electrical activity of the heart during climbing (Figure 3, Table 1). The sensors consist of electrocardiogram (ECG) electrodes placed in contact with the skin to measure small electrical changes from cardiac muscle depolarization and repolarization during each cardiac cycle (heartbeat). The continuous ECG measurements are used to determine heart activity parameters (e.g., heart rate. heart rate variability). 

During indoor lead climbing, Breen et al. used the Hexoskin biometric compression shirt (Table 1) to measure continuous heart rate [14]. The shirt contains three textile electrodes (two at the thoracic level, one at the abdominal level) to yield three-lead ECG recordings at 256 Hz, which are used to derive the heart rate every 1 s. During a lead climb, the heart rate showed large variability with a range between 70 and 174 beats/min, and a median of 160 beats/min (Figure 4). Using time-matched climbing video, the heart rate showed decreases when resting on the wall, and remarkably large short-term increases (70–172 beats/min) when ascending a difficulty section of the climbing route that required a climber to try different strategies (e.g., variations in hold sequences, body movements). Villar et al. showed a mean absolute error of 1% for the heart rate using the Hexoskin shirt during moderate- and high-intensity exercise [16]. 

During a simulated indoor bouldering competition, Nishitani et al. used an ECG sensor embedded in a chest band (RC3 GPS, Polar, Kempele, Finland) to measure continuous heart rate [31]. Perceived exertions were measured after climbing. The high-ranking climbers, which indicated low perceived exertion, rapidly reached a consistent peak heart rate (approximately 188 beats/min) while climbing each of the five boulders. 

### 3.4. Eye Gazing Sensors

**Figure 5:**
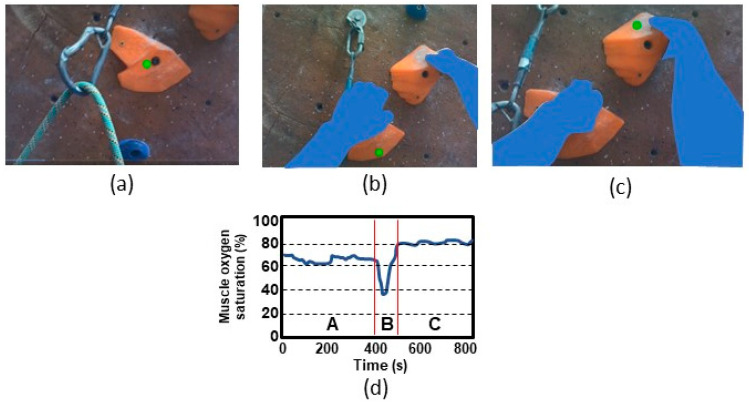
Examples of data from two types of sensors: eye gazing sensors (**a**–**c**), and NIRS skeletal muscle sensor (**d**). Eye gazing data during indoor lead climbing show the climber’s gaze location (green dot) overlaid on a sequence of three images from the scene video camera of eye tracking glasses. The first and second images (**a**,**b**) show a climber maintaining their eye gaze on the same hand hold. The third image (**c**) shows the climber shifting their gaze to the next hand hold. The NIRS sensor data (**d**) show moderate (60–70%), low (35%), and high (80–85%) muscle oxygen saturation during moderate-intensity (Period A), high-intensity (Period B), and low-intensity (Period C) muscle exertion, respectively.

Eye gazing sensors are wearable eye tracking glasses (ETG) used to measure the eye gazing behavior (Figure 3, Table 1). Eye gazing behavior is a critical aspect of a climber’s visual—motor skill, which is the ability to perceive relevant optical information from the climbing environment (e.g., surface texture and inclination, potential paths, places to avoid) and to effectively coordinate body movements to achieve an outcome (e.g., ensure contact with climbing holds or surfaces with appropriate timing, velocity, force, spatial orientation). Wearable ETG are mobile systems that continuously measure eye movement displacements to determine gaze locations in a scene video (Figure 5). The ETG consists of: (1) left and right eye-facing cameras embedded within an eyeglasses frame that continuously measures the location of the pupil and reflection of the cornea to determine eye movement displacements; and (2) a front-facing scene camera also embedded within the eyeglasses frame. After the eye movement displacements are calibrated by fixating on known locations in the scene video, the climber’s gaze locations are overlaid in the scene video. 

ETG can be applied before climbing (i.e., route-preview) and during climbing. For route-preview, Grushko et al. used ETG (SMI, SensoMotoric Instruments Inc, Teltow, Germany) to examine the visual search strategies of participants as they previewed lead climbing routes in a climbing gym [32]. The gaze behavior was used to determine the time spent performing four different route previewing strategies with increasing levels of complexity: fragmentary, ascending, zigzagging, sequence-of-blocks. The study found that climbers use route previewing strategies of varying complexity for different purposes (e.g., simple preview to identify locations of potential holds, more sophisticated preview to plan sequence of movements). The study also showed that skilled climbers use route preview to mentally rehearse climbing movements and spend most time examining potentially difficult sections of the route (i.e., cruxes). Seifert et al. used ETG (SMI, SensoMotoric Instruments Inc, Teltow, Germany) to measure the gaze behavior of experienced and inexperienced climbers as they previewed a top rope climbing route in a climbing gym [20]. The study showed that the number of scan paths during route preview was positively correlated with duration of exploration while climbing. Additionally, longer durations in performing more sophisticated previews were associated with longer durations of immobility (i.e., no body movement) during climbing. 

During climbing, Hacques et al. used ETG (Tobii Pro Glasses 2, Tobii, Stockholm, Sweden) to examine the effect of practice on gaze patterns towards the handholds [8]. Dupuy et al. used ETG (Eye Mark Recorder IV, Nac Image Technologies, Salem, MA, USA) to measure eye movements of experienced climbers while attempting the same set route in three conditions: on-sight (no prior knowledge of the route), after repetition (fifth attempt), and maximal speed to assess the influence of time pressure [33]. Nieuwenhuys et al. used ETG (Model 501, Applied Systems Laboratories, Huntsville, AL, USA) to track gaze locations of participants with no climbing experience while top rope climbing two identical horizontal routes (i.e., traverses), which were built on high (4.25 m) and low (0.44 m) levels above the floor along a vertical indoor climbing wall to provide high and low anxiety conditions, respectively [10]. This study showed a decrease in route exploration during climbing as anxiety increased, which provided evidence for a decrease in processing efficiency of perceptual-motor tasks. 

### 3.5. Skeletal Muscle Characterization Sensors

Wearable surface electromyography (EMG) and near-infrared spectroscopy (NIRS) sensors measure the activity and functional status of localized muscles (e.g., forearm flexor muscles) during climbing (Figure 3, Table 1). Wearable EMG and NIRS are non-invasive sensors used to assess muscle status based on their electrical and hemodynamic behavior, respectively. These sensors provide continuous measurements of localized muscle during climbing, whereas other invasive techniques, such as lactate measurements from blood samples, only provide the muscle status at the moment of the sample collection (i.e., before or after climbing). 

The EMG sensors consist of electrodes placed in contact with the skin to measure the electrical potential from neural activation of localized skeletal muscles near the electrode site. The continuous EMG measurements are used to determine muscle activity parameters (e.g., muscle activation timing, muscle force based on magnitude of EMG signal, muscle fatigue based on decreases in the median frequency of a power spectrum analysis). 

During climbing, Watts et al. used an EMG sensor system (Tel-100, BioPac Systems, Goleta, CA, USA) [34] to measure electrical activity of finger flexors, and showed constant activation of the forearm musculature during contact with holds, and greater muscle fiber recruitment than during maximum handgrip dynamometry [25]. These results suggest that more eccentric (i.e., lengthening of muscle) contraction is applied during climbing to maintain contact with a hold and resist changes in finger positions, whereas more concentric (i.e., shortening of muscle) contraction is applied during handgrip testing. This demonstrates the importance of using wearable sensors for muscle activity measurements during climbing, as compared to traditional methods (e.g., hand dynamometer). 

The NIRS sensors consist of near-infrared light sources and detectors placed in contact with the skin to measure light absorbance of local skeletal muscles near the device. The continuous NIRS measurements are used to determine muscle oxygen saturation (i.e., oxygen consumption; Figure 5). Thus, the sensors provide a method to examine local muscle oxygen responses as compared to systemic oxygen responses as measured from respiration sensors. The NIRS parameters determined are local muscle oxy-hemoglobin (oxy-HB), deoxy-hemoglobin (deoxy-HB), total hemoglobin (total-HB), and muscle oxygen saturation (oxy-HB/total-HB) relative to the blood volume under the device. For muscle oxygenation metabolism, deoxy-HB reflects the balance between O_2_ delivered and O_2_ consumption, and thus provide an indicator of the physiological status and performance of a muscle.

Climbing requires high-intensity forearm finger flexor muscle contractions which induce repeated periods of local ischemia separated by short periods of reperfusion [26]. These contractions require both oxidative (i.e., aerobic) and non-oxidative (i.e., anaerobic) metabolic pathways. During climbing, NIRS has been used to determine local muscle oxygen responses. Balas et al. used a wearable NIRS device (Portamon, Artinis Medical Systems, BV, Zetten, The Netherlands) to measure oxygen saturation of the finger flexor muscles during climbing [26,35]. Results showed that more advanced climbers have greater oxygen desaturation of the finger flexors during sustained and intermittent contractions. 

## 4. Integration of Multiple Sensors with Climbing Video for Location-Specific Analysis

The sensors described above provide time–course biometric data during climbing, but do not provide spatial information about the location of the climber relative to spatially varying characteristics of the climbing route (e.g., inclination of wall, setting of holds). Breen et al. developed a micro-location biometric system (MLBS) to integrate continuous data from three types of wearable sensors (heart, respiration, body movement) and one external sensor (video), with a customized visualization and analysis system [14]. The system provides three physiological metrics (heart rate, breathing rate, minute ventilation rate) and one body movement metric (hip acceleration) that are combined with the corresponding video frame to determine location-specific biometrics. 

The MLBS also addresses the challenge of synchronizing, visualizing, and analyzing the large multi-dimensional information that are simultaneously collected from multiple sensors during climbing. The system first time-matches the four biometrics with a climbing video, and then provides an interactive visualization tool that displays each biometric time-course data with a moving time-marker corresponding to the displayed video frame. The MLBS also provides an analysis tool to rapidly compare the temporal characteristics of multiple biometrics at different locations along the climbing route.

## 5. Discussion

Table 2 provides a detailed guide for sensor selection by describing the practical benefits, limitations, and relative cost for each type of sensor. For body movement metrics, the three main types of sensors with increasing complexity are standalone accelerometers, IMU, and 3D body motion capture systems using multiple external cameras. For respiratory sensors, face mask-based metabolic systems are substantially more burdensome and expensive, as compared to a chest band-based RIP systems, but provide more comprehensive biometrics with direct measurements of airflow and respiratory gases, whereas RIP systems, such as Hexoskin, are more practical for climbing applications with no impact on climbing performance. For heart activity metrics, the sensor technologies are highly accurate, comfortable to wear, and lower cost, but potentially limited in terms of climbing applications. To measure eye gazing behavior, eye tracking glasses can provide practical information about route reading and exploring abilities, but have substantial cost. Finally, for skeletal muscles metrics, surface EMG and NIRS sensor technologies can provide non-invasive and feasible methods to continuously measure changes in the local muscle (e.g., forearm) response during climbing, but data interpretation can be complex. 

### 5.1. Body Movement Sensors

Body movement measurements have several practical climbing applications. Movement is a critical aspect of climbing and performance metrics (Table 3) related to movement can be determined using wearable sensors (e.g., accelerometers), external scene cameras, and embedded force sensors within holds on climbing walls. Key performance metrics derived from body movement (i.e., fluidity metrics) include geometric index of entropy, jerk coefficient, time spent at different climbing states (e.g., immobility), body’s center of mass locations, and load distribution along climbing path.

Fluidity is an indicator of the ease and efficiency of movement during climbing, which is a key indicator of climbing skill [5]. Fluidity metrics can be derived from different parameters including geometric index of entropy (spatial parameter), hip jerk coefficient (spatio-temporal parameter), and immobility (temporal parameter). Geometric index of entropy is the movement of the center of mass from an ‘ideal’ trajectory defined by a mathematically derived convex hull [36]. The geometric index of entropy can be used as an indicator of climbing performance, method to differentiate competing climbing strategies [37], and metric for climbing fluidity and energy expenditure [38]. Hip jerk coefficient includes the 3D spatial and temporal dimensions based on hip acceleration [12]. Immobility is the ratio of time spent in two states: movement versus lack of movement at the center of mass [39].

Time spent at different climbing states (e.g., resting/ascending ratio) and body orientations (e.g., facing wall/side into wall) can be determined from body movement measurements and serve as indicators of climbing performance. Boulanger et al. used IMU sensors to determine four different climbing states based on hip and limb movements, which include: immobility (all points immobile), postural regulation (pelvis movement, limbs immobile), hold interaction (pelvis immobile, at least one limb moving), and traction (pelvis and at least one limb moving simultaneously) [19]. Orth et al. used an external scene camera to derive hip positions and determine hip jerk, immobility, and geometric index of entropy [40]. These metrics were used to monitor the effect of practice on the climbing skill conditions of body orientations—facing wall and side on wall [40]. Seifert et al. used IMU sensors to determine limb and body orientations, which can be good indicators of climbing performance [21].

Indoor climbing walls with holds embedded with force sensor can be used to determine the mechanics of climbing [41]. Measurements from a 3D force sensor on each hold, which are normalized by the climber’s weight, can provide feedback on climbing technique and power. The applied forces from the feet and hands can be visualized with plots of the body’s center of mass location along the climbing path to evaluate load distribution. A climbing route with several or a complete set of instrumented holds can be used to analyze the efficiency and correctness of the climber’s movements.

### 5.2. Respiration Sensors

The respiration sensors can determine various breathing parameters during climbing, which include breathing rate, tidal volume, and minute ventilation. Breathing rate can be applied for climbing as an indicator of perceived exertion and fatigue (Table 3). Nicolo et al. showed that breathing rate, as compared to other biometrics (i.e., heart rate, oxygen consumption, blood lactate), is strongly associated with perceived exertion during exercise [9]. Breathing depth based on tidal volume or minute ventilation can be used as an indicator of breathing quality during climbing. Studies have shown that deeper breathing from greater expansion of the diaphragm can indicate a more well-trained athlete [42].

Breathing sensors can monitor interventions based on the relationship between a climber’s respiration and factors that could be associated with a climber’s overall performance level. These climbing-specific factors associated with respiration parameters include the following: movement [24], hold inclination [43], stress [44], speed of ascent [45,46], and active recovery [47]. Balas et al. found a significant relationship between climbing ability and oxygen consumption [24]. Climbers with higher ability had lower oxygen consumption, which indicates a greater economy of movement (i.e., climbing economy). Therefore, oxygen consumption may provide be a good indicator of climbing economy, which leads to increased time to exhaustion and improved climbing performance.

Climbing-specific endurance is determined by both systemic and local muscle oxygen kinetics [26,48]. For climbing, studies indicate that systemic oxygen demand has a stronger association with the aerobic energy system used for large muscle contractions (e.g., legs) and climbing at higher speeds, and less associated with high-intensity small muscle (e.g., finger flexors) contractions [49]. Watts et al. describes that local conditions with decreases in oxygen and increases in blood lactate during sustained small muscle contractions do not stimulate systemic oxygen responses [6]. Additionally, aerobic demand in climbing may be intermittent due to the intermittent body movements and finger flexor contractions during climbing, as compared to the continuous movements during running and cycling [50]. Therefore, breathing parameters for both systemic oxygen consumption and local muscle oxygen conditions can be important indicators of climbing endurance.

### 5.3. Heart Activity Sensors

Heart rate, which is one of the easiest physiological parameters to measure, increases substantially during climbing. Studies measured peak heart rates of 88–93% at maximum, and lead climbs tend to have higher minimum and average heart rates but not necessarily higher peak values, as compared to bouldering [26,51,52,53]. Heart rate increases with greater climbing speed [54] and the level of difficulty of climbing route, which is likely due to more time spent in isometric body positions [7].

Heart rate can increase during climbing due to several factors unrelated to climbing intensity, which include placing arms above heart, fear of falling, fear of heights, and stress during climbing competitions [7,55,56]. Some of these factors, especially the psychological factors, cannot be reliably measured.

**Table 3:** Climbing performance metrics based on parameters derived from sensor measurements.

Climbing Skill/ Performance Metric	Sensor-Derived Parameter	Body Movement Sensor (Location)	Respiration Sensor	Heart Sensor	Eye Gazing Sensor	Skeletal Muscle Sensor	External Scene Camera	Hold-Embedded Force Sensor	Reference
Recovery rate during rest/climb patterns	Spatial variations in heart rate, systemic oxygen uptake	ACC (hip)	BR, MV	HR	-	-	Yes	-	[14]
Climbing fluidity	Jerk coefficient, immobility, geometric index of entropy	ACC (hip), IMU (hip, legs, forearms)	-	-	-	-	-	-	[5,12,36,37,38,39]
Time spent at different climbing states	Resting/ascending ratio, body orientations	ACC (hip), IMU (hip)	-	-	-	-	Yes	-	[19,21,40]
Load distribution along climbing path	Location of center of mass	-	-	-	-	-	Yes	Yes	[41]
Perceived exertion	Breathing rate, breathing depth	-	BR, MV	-	-	-	-	-	[9,42]
Climbing economy, aerobic energy demand	Systemic oxygen consumption	-	MV	-	-	-	-	-	[24]
Cardiac modulation of nervous system	Heart rate variability	-	-	ECG	-	-	-	-	[57]
Time spent at different gaze behavior states during climbing	Exploring ahead versus maintaining focus	-	-	-	Gaze behavior	-	-	-	[8]
Route previewing strategy and skill	Identify visual strategies: ascending, fragmentary, zigzagging, sequence-of-blocks	-	-	-	Gaze behavior	-	-	-	[32]
Forearm muscle endurance, fatigue	Local muscle oxygenation, muscle activation	-	-	-	-	NIRS, EMG (forearm)	-	-	[11,48,58,59]

BR breathing rate, MV minute ventilation, HR heart rate, ECG electrocardiogram, ACC accelerometer, IMU inertial measurement unit, EMG electromyography, NIRS near-infrared spectroscopy.

In addition, oxygen demand is mainly localized to small muscles (e.g., forearm muscles) during climbing, which does not significantly impact heart rate. Therefore, heart rate may not be a reliable indicator of climbing intensity (e.g., perceived exertion level) [60]. Furthermore, heart rate can increase significantly greater than oxygen consumption during climbing [43,61]. Thus, heart rate may not be a reliable indicator of climbing aerobic demand.

Heart rate may also not reflect climbing fatigue. During climbing, the heart muscle is never fully loaded [60]. Unlike cardio-intensive sports (e.g., running, cycling, ice hockey), the large muscles (e.g., leg muscles) are only partly activated, while the small muscles (e.g., forearm muscles) are fully activated during bouldering and lead climbing. Small muscles fatigue rapidly due to increased blood lactate, which leads to muscle coordination failure and climber falling [54,62]. Therefore, local exhaustion of the arm muscles, and not the general exhaustion of the entire climber, is the limiting factor. Furthermore, heart rate can quickly recovery between boulders during competitions, and may therefore not be a reliable indicator of fatigue accumulated from multiple climbs [51].

ECG measurements can be used to determine heart rate variability (HRV), which may have climbing applications (Table 3). HRV is a measure of the variation in time between heartbeats, which reflects cardiac modulation of the autonomic nervous system by indicating the balance between sympathetic (intense fight-or-flight) and parasympathetic (relaxing body and mind). Higher HRV indicates a more relaxed state and correlates with better health, resilience, and increased fitness. Lower HRV indicates a stress and fatigued nervous system. HRV can be used to monitor positive and negative trends in fitness, fatigue, and readiness to compete. In climbing, HRV could be used to help track the effectiveness of high intensity interval training interventions (i.e., increases in HRV), and to help identify overtraining (i.e., decreases in HRV) [57]. In climbing, HRV may allow athletes and coaches to identify adaptations to interventions designed to conserve energy. Tracking HRV may be more important for lead climbing, where the use of energy system interventions may take on greater significance when compared to the bouldering discipline.

### 5.4. Eye Gazing Sensors

Eye gazing data have various practical applications during climbing and during route-preview (Table 3). During climbing, key performance metrics include (1) gaze complexity (i.e., visual entropy), (2) gaze behavior states of proactive gaze (looking ahead) versus online gaze (maintaining focus) for an area of interest around a handhold, and (3) gaze attention (i.e., maintaining focus versus looking ahead) [8]. Hacques et al. applied these performance metrics to determine the effect of practice on the skill of using visual information to identify action opportunities and create movement in climbing [8]. Visual entropy can be used as an indicator of goal-directed visual performance and visual certainty/uncertainty [8].

Eye gazing sensors can be used to determine a climber’s strategy and skill level for route previewing, which can enhance climbing fluency and performance. Visual inspection of a route before climbing (route previewing) can allow the climber to perceive opportunities for action by the surface and layout, and thus to minimize misperception. The importance of route previewing has been shown in several studies [10,63,64]. Sanchez et al. showed that climbers using route preview had improved climbing form performance with fewer and shorter-duration stops during climbing [63]. Measuring gaze behavior during route preview can be used to identify four different visual strategies for route previews: ascending, fragmentary, zigzagging, and sequence-of-blocks [32]. Simpler strategies (i.e., ascending, fragmentary) are used to find the overall route, whereas more sophisticated strategies (i.e., zigzagging, sequence-of-blocks) are associated with deep visual inspection. The gaze behavior can be used with a classification method to automatically determine the time spent performing each of the four visual strategies [20]. Seifert et al. showed that the more sophisticated strategies were associated with longer durations of immobility (i.e., no body movement) during climbing, supporting the need for a deeper visual analysis during previewing when the body movements between certain holds could not be clearly perceived [20]. For climbers, this method could be used to help improve route previewing and mental imagery skills, and subsequent climbing performance.

### 5.5. Skeletal Muscle Characterization Sensors

For climbing, wearable NIRS and EMG sensors are critical to measure the activity and functional status of the forearm finger flexor muscles. The finger flexors are a critical and often limiting factor for climbing performance. They are often the first muscles to become fatigued due to long-term isometric contractions. During climbing, these muscles can become oxygen-deprived due to muscle contraction-induced ischemia (i.e., insufficient blood supply). Since the aerobic energy pathway will be limited due to localized ischemia, the energy needed to sustain the flexor muscle contraction is produced by the anaerobic energy pathway. Anaerobic metabolism produces lactate that can create local acidic conditions, which decrease muscle contraction forces, induce muscle fatigue, and create sensations of tightness and pain in the forearm muscle, and loss of muscle coordination.

NIRS sensors can be applied during climbing to monitor forearm muscle oxygen desaturation and recovery for different climbing strategies (Table 3) [11,48]. These sensors are especially useful due to their ability to continuously monitor local oxygen levels within the working muscles, which are often the limiting factor for climbing endurance and fatigue. In addition, monitoring the local muscle oxygenation using NIRS and the systemic oxygen consumption using respiration sensors is critical since both central (or systemic) and peripheral (or local) systems can impact climbing performance [26].

NIRS can be used to monitor the time to half-recovery (T_1/2_) between muscle contractions as an indicator of tactical recovery ability [58]. These sensors can also be used to monitor muscle oxygen breakpoint (MOB) as an indicator of aerobic capacity [26]. Additional applications include monitoring T_1/2_ and MOB while manipulating different tactical variables (e.g., intermittent or rhythmic contraction-relaxation patterns, increasing-decreasing load patterns) or tracking the effectiveness of different training interventions (e.g., continuous climbing, high-intensity interval training, sprint-interval training).

EMG sensors can be applied during climbing to monitor forearm muscle activity as an indicator of applied force for different climbing techniques (Table 3) [59]. These sensors can also be used to monitor muscle activity for different climbing strategies (e.g., foot–hip techniques, climb–rest patterns) and to track different training interventions (e.g., muscular development).

## 6. Future Applications

The tremendous advances in new wearable sensors and their application in climbing will support training and performance improvement. The increased use of sensors during climbing allows for a better understanding of the specific and potentially different needs (e.g., sensor types, biometrics, performance indicators) for each type of climbing (e.g., bouldering, lead, speed climbing). In the future, large volumes of data collected from sensors during climbing can also be combined with artificial intelligence (AI) technologies to identify patterns in climbing performance. Sensors integrated with AI can be used as coaches to direct climbers for optimal training and performance.

## 7. Conclusions

Advances in wearable and non-invasive sensor technologies, along with the recent surge in popularity of rock climbing, has generated a tremendous desire and opportunity to maximize performance by analyzing sensor data collected during climbing. Without sacrificing performance, these types of sensors enable the continuous measurement of body movements and physiologic responses while ascending the climbing wall in order to obtain this critical information. We demonstrated the capabilities and potential applications of five types: body movement, respiration, heart activity, eye gazing, and skeletal muscle characterization. This review provides details of the practical benefits, limitations, and cost of each sensor type (Table 2) in support of determining appropriate sensors for specific climbing applications, allowing the enhancement of training and strategies.

## Figures and Tables

**Table 2:** Practical benefits and limitations of wearable sensors.

Sensor Type	Example Product (Company), Cost	Practical Benefits	Practical Limitations
Body movement—accelerometer	Hexoskin (Carre Technologies, Montreal, QC, Canada), medium cost	Comfort, time-matched heart rate, respiration	Only linear movement since no gyroscope (rotational movement), need body-specific shirt size
Body movement—IMU (accelerometer, gyroscope, magnetometer)	MotionPod (Movea, Grenoble, France), low cost	Body motion animations to visualize movements	Potential drift errors
Body movement—insole pressure distribution	Pedar-X (Novel, Munich, Germany), medium cost	Practical method to measure forces applied to holds	Complexity of data analysis with continuous 2D spatial maps of applied forces
Body movement—motion capture system (MCS)	Mac 3D System (Motion Analysis, Santa Rosa, CA, USA), high cost	Completely non-invasive since no sensors are worn, only external cameras used	Complexity of system setup with multiple synchronized external camera
Body movement—force sensor embedded within climbing hold	K3D120 triaxial force sensor (ME-Meβsysteme, Hennigsdorf, Germany), medium cost	Completely non-invasive since no sensors are worn	Need to build a customized climbing wall.
Respiration—airflow, O_2_, CO_2_	METAMAX 2B (Cortex, Biophysik, GmbH, Leipzig, Germany), high cost	Direct measurement method to determine breathing metrics	Discomfort during vigorous activity, potential negative impact to performance with face mask
Respiration—respiratory inductive plethysmography (RIP)	Hexoskin (Carre Technologies, Montreal, QC, Canada), medium cost	Comfort, collects complementary heart, accelerometer data	Indirect measurement method to determine breathing metrics
Heart activity—electrocardiography (ECG)	Hexoskin (Carre Technologies, Montreal, QC, Canada), medium cost	Comfort, collects complementary respiration, accelerometer data	Uses a compression shirt, as compared to simpler chest band (e.g., Polar)
Heart activity—electrocardiography (ECG)	Polar (Polar Electro OY, Kempele, Finland), low cost	Simple, comfortable design with single chest band	No complementary data such as time-matched respiration, movement data (e.g., Hexoskin)
Eye gazing—eye tracking glasses (ETG)	Tobii Pro Glasses 2 (Tobii, Stockholm, Sweden), high cost	Unique ability to determine and practice route reading skills	Eye frames may slightly reduce field of view while exploring route during climbing
Skeletal muscle electromyography (EMG)	Tel-100 System (BioPac Systems Goleta, CA, USA), low cost	Unique ability to determine local muscle electrical activity	Possible measurement uncertainty due to artifacts from activity of nearby muscles
Skeletal muscle—near infrared spectroscopy (NIRS)	PortaMon (Artinis Medical Systems, BV, Zetten, The Netherlands), low cost	Unique ability to determine local muscle oxygen response, which complements whole-body oxygen response using respiration sensors	Possible measurement uncertainty due to variations in tissue arrangement between skin surface and muscle of interest

## Data Availability

Not applicable.
